# Power-injectable peripherally inserted central catheters in intensive care patients

**DOI:** 10.1186/cc10816

**Published:** 2012-03-20

**Authors:** MG Annetta, C Marano, A Brutti, D Celentano, M Pittiruti

**Affiliations:** 1 Catholic University, Rome, Italy

## Introduction

In ICUs, peripherally inserted central catheters (PICCs) may be an alternative option to standard central venous catheters, particularly in patients with coagulation disorders or at high risk for infection. Some limits of PICCs (such as low flow rates) may be overcome by the use of power-injectable catheters.

## Methods

We have retrospectively reviewed all of the power-injectable PICCs inserted in adult and pediatric patients in the ICU during a 12-month period, focusing on the rate of complications at insertion and during maintenance. All PICCs were inserted by specifically trained nurses, using ultrasound guidance and the microintroducer technique, according to a specific insertion protocol.

## Results

We have collected 89 power-injectable PICCs (65 in adults and 24 in children), 4 to 6 Fr, both multiple and single lumen. All insertions were successful. There were no major complications at insertion and no episodes of local infection or catheter-related bloodstream infection. Noninfective complications during management were not clinically relevant. There was one episode of symptomatic thrombosis during the stay in the ICU and one episode after transfer of the patient on a nonintensive ward.

## Conclusion

Power-injectable PICCs have many advantages in the ICU: they can be used as multipurpose central lines for any type of infusion including high-flow infusion, for hemodynamic monitoring, and for high-pressure injection of contrast media during radiological procedures. Their insertion is successful in 100% of cases and is not associated with significant risks, even in patients with coagulation disorders. Their maintenance is associated with an extremely low rate of infective and noninfective complications.

